# The effect of ticagrelor on coronary microvascular function after PCI in patients with ACS compared to clopidogrel: A systematic review and meta-analysis

**DOI:** 10.1371/journal.pone.0289243

**Published:** 2023-08-29

**Authors:** Xiaohan Qiu, Xiaohui Li, Kang Fu, Wentao Chen, Wenqiang Chen

**Affiliations:** Department of Cardiology, National Key Laboratory for Innovation and Transformation of Luobing Theory, The Key Laboratory of Cardiovascular Remodeling and Function Research, Chinese Ministry of Education, Chinese National Health Commission and Chinese Academy of Medical Sciences, Qilu Hospital of Shandong University, Jinan, China; Saud Al-Babtain Cardiac Centre, SAUDI ARABIA

## Abstract

**Background:**

The function of coronary microcirculation is an important factor in predicting the prognosis of patients with acute coronary syndrome (ACS) who receive percutaneous coronary intervention (PCI) therapy. Ticagrelor, a type of oral P2Y12 inhibitor, is widely prescribed to ACS patients and can improve prognosis compared to clopidogrel. However, the efficacy of ticagrelor on coronary microcirculation, compared to clopidogrel, remains unclear. The objective of this meta-analysis was to determine the efficacy of ticagrelor on coronary microcirculation.

**Methods:**

The PubMed, Cochrane Central Register of Controlled Trials (CENTRAL), and ClinicalTrials.gov databases were comprehensively searched to identify studies until November 2022. Data was pooled using the fixed effects model or random effects model based on the level of heterogeneity. Sensitivity analyses were performed to measure the effects of potential confounders.

**Results:**

After screening, 16 trials with a total of 3676 participants were ultimately included in the analysis. The meta-analysis revealed that compared to clopidogrel, patients receiving ticagrelor exhibited a more significant reduction in the IMR (WMD: -6.23, 95% CI: -8.41 to -4.04), a reduction in the cTFC (WMD: -1.88; 95% CI: -3.32 to -0.45), and greater increases in CFR (WMD: 0.38; 95% CI: 0.18 to 0.57), MBG (RR 1.29, 95% CI 1.12 to 1.48), and TIMI (RR 1.03, 95% CI 1.00 to 1.06).

**Conclusion:**

Our findings suggest that, compared to clopidogrel, ticagrelor has a significant effect in reducing coronary microcirculatory resistance, enhancing coronary blood flow reserve, and improving myocardial perfusion.

## Introduction

Acute Coronary Syndrome (ACS), as a crucial component of coronary heart disease (CHD), has become a significant cause of human mortality worldwide. Percutaneous Coronary Intervention (PCI) is widely acknowledged as one of the most effective approaches for restoring blood flow in cases of acute myocardial infarction or unstable angina. When compared to traditional medical treatment, PCI has demonstrated superior efficacy in reinstating myocardial perfusion, reducing myocardial ischemia or infarct size, and improving clinical outcomes [1]. Despite the continuous maturity and improvement of PCI technology, there are still some patients who do not benefit from PCI treatment. Even after the anatomically perfect restoration of the epicardial occlusion/stenosis, clinical evidence indicates that revascularization does not always re-establish effective microcirculatory perfusion [2]. Coronary microcirculatory dysfunction is not only a major cause of myocardial ischemia in patients with "normal or near-normal" findings on coronary angiography but also a significant contributor to the impaired quality of life and adverse prognosis following coronary revascularization surgery [3–5].

Dual antiplatelet therapy (DAPT) is the mainstay of pharmacotherapy for patients with ACS receiving PCI therapy [1]. Recent clinical studies have shown that ticagrelor has a better effect on the short-term prognosis of patients with ACS who undergo PCI than clopidogrel [6]. Pharmacodynamic studies have demonstrated that platelet inhibition by ticagrelor is greater and faster than that by clopidogrel [7]. A strong antiplatelet effect can inhibit thrombus microembolization and benefit microvascular function [8,9]. Moreover, compared to clopidogrel, ticagrelor can inhibit adenosine degradation, prolong the biological half-life of adenosine and increase the vasodilatation effects of adenosine in both endogenous and exogenous pathways [10]. Adenosine improves coronary microcirculation by improving endothelial function and reducing the inflammatory response [11]. Based on its pharmacological mechanism of action, there may be a benefit for microcirculatory lesions, but clinical studies are not yet conclusive.

Several clinical trials have investigated the effect of ticagrelor on coronary microvascular function. However, these studies have reported conflicting results about whether ticagrelor improved coronary microcirculation. Several studies have suggested that ticagrelor ameliorates coronary microcirculation [12–14]. In contrast, other studies demonstrated that ticagrelor exhibited no additional beneficial effect on coronary microvascular function [15,16]. To validate the effect of ticagrelor on coronary microcirculation, we conducted the current systematic review.

## Methods

### Protocol and guidance

This study was conducted in accordance with the Preferred Reporting Items for Systematic Reviews and Meta-Analysis (PRISMA) guidelines [17]. The protocol for this review was registered with PROSPERO (CRD42022375073).

### Inclusion criteria

We considered studies to be eligible for inclusion if they enrolled patients with ACS who were undergoing PCI; if they compared ticagrelor at any dose with clopidogrel; if they provided information on common parameters of CMD; and if they were randomized controlled trials (RCTs).

### Exclusion criteria

We excluded trials if they were review articles, case reports, animal experiments, or observational studies; if all the participants received ticagrelor; if they enrolled patients with CMD rather than patients with ACS treated with PCI; or if we could not extract data from the study.

### Primary outcomes and secondary outcomes

The primary outcome was a reduction in levels of the index of microvascular resistance (IMR) and an increase in coronary flow reserve (CFR). Secondary outcomes were increases in myocardial blush grade (MBG) and thrombolysis in myocardial infarction flow grade (TIMI) and reductions in corrected TIMI frame count (cTFC).

Index of microvascular resistance (IMR) is a new index recently proposed to evaluate the microvascular function distal to a stenotic lesion, which is defined as a ratio of distal coronary pressure to distal coronary flow, i.e., the product of the distal coronary pressure (Pd) and mean transit time(T) of a saline bolus during maximal hyperemia [18]. Pd and T are measured by a pressure guidewire equipped with a temperature sensor. IMR is independent of subepicardial vascular function and able to specifically evaluate microvascular function with a good reproducibility [18,19]. CFR, which reflects the vasodilator capacity of the coronary microcirculation, can be calculated by dividing hyperemic flow by resting flow corrected for the rate–pressure product [20]. It is an overall indicator of the reserve function of the entire coronary system. Coronary microvascular function is frequently assessed by evaluating the coronary microvascular response to vasodilators, because of the limitation of current imaging techniques in displaying morphological changes of coronary microvasculature. CFR is a commonly used index [21,22].

“Slow coronary flow” is an angiographic phenomenon characterized by a delayed visualization of distal vessels of a non-obstructive coronary artery, which is considered a manifestation of CMVD. The definition for “slow coronary flow” varies between different studies, some using TIMI flow grades 1–2 while others using a modified TIMI flow count of >25 frames [23]. TIMI (grades 0 to 3) is only a semi-quantitative parameter and cannot reflect coronary microvascular function [24]. cTFC is the number of frames from the beginning of coronary artery imaging to the standardized distal marker imaging, which overcomes the shortcomings of semi-quantitative nature of TIMI flow grading. However, it does not directly reflect coronary microvascular flow [25]. In summary, IMR and CFR are quantitative and widely available indicators for assessing coronary microvascular function. Therefore, IMR and CFR are chosen as the primary outcome measures, while the other indicators serve as secondary outcome measures.

### Data sources and search strategies

From inception to 12 November 2022, we comprehensively searched the PubMed, Cochrane Central Register of Controlled Trials (CENTRAL), and ClinicalTrials.gov databases for eligible RCTs using the following keywords: (ticagrelor) OR (Brilique) OR (AZD-6140) OR (Brilinta) OR (3-(7-((2-(3,4-Difluorophenyl)cyclopropyl)amino)-5-(propylthio)-3H-(1–3)-triazolo(4,5-d)pyrimidin-3-yl)-5-(2-hydroxyethoxy)cyclopentane-1,2-diol) AND (Microvascular) OR (Microcirculation) OR (coronary artery) OR (coronary microvascular dysfunction) OR (CMVD) OR (CMD) AND (randomized controlled trials) OR (RCT) (S1 Table).

### Study selection

After removing duplicates, two authors independently screened titles and abstracts and subsequently full-text articles. Disagreements were resolved by consensus.

### Data collection process

Two independent researchers (QXH and LXH) extracted data with a standard data extraction form from the included trials. Data include study characteristics (first author, publication year, country, duration of medication, and number of patients and outcomes reported) and patient characteristics (mean age in years, percentage of men). If randomized controlled trials had more than two groups, we pooled data from the separate treatment arms. Disagreements were resolved by consensus. All data extractions were completed by two reviewers (QXH and LXH) and checked by another reviewer (FK and CWT).

### Assessment of risk of bias and quality of evidence

Two researchers independently assessed the methodological quality of the included studies using the Cochrane Risk of Bias Tool [18]. The six specific areas in which the Cochrane risk of bias tool was objectively evaluated include the generation of randomized sequences, concealment of allocation protocols, blinding of study participants and related persons, blinding of outcome evaluators, incomplete data on study results, and selective reporting of results. Potential publication bias was assessed by visualization of asymmetry in funnel plots (when there were at least 10 studies reporting the outcome) or by Egger’s test and Begg’s test.

### Data synthesis and analysis

Changes in IMR, CFR, MBG, TIMI grade III and cTFC were used to assess the efficiency of ticagrelor on coronary microvascular function, determining the difference between the intervention and control groups with mean and risk ratio. Continuous variables were used to analyze the weighted mean difference (WMD) with the 95% CI effect size. Dichotomous outcomes were used to analyze the relative risk (RR) with a confidence interval (CI) of 95%. Statistical heterogeneity was assessed using the chi‐squared test (p < 0.10 was considered statistically significant for heterogeneity) and was quantified using the I^2^ statistic (0–25% low heterogeneity, 25–50% moderate heterogeneity, 50–75% substantial heterogeneity, 75–100% high heterogeneity) [26]. We used the fixed effects model or the random effects model to perform the meta-analysis based on the I^2^ values. STATA 17.0 software (Stata Corporation, Texas, USA) was used for data analyses.

### Sensitivity analyses

Where there was a high level of statistical heterogeneity, we conducted sensitivity analyses by excluding trials with a high or unknown risk of bias. Based on the sensitivity analysis results, eliminating each study one at a time sequentially did not substantially influence any of the assessed factors.

## Results

### Study selection, characteristics, and quality assessment

**Fig 1:**
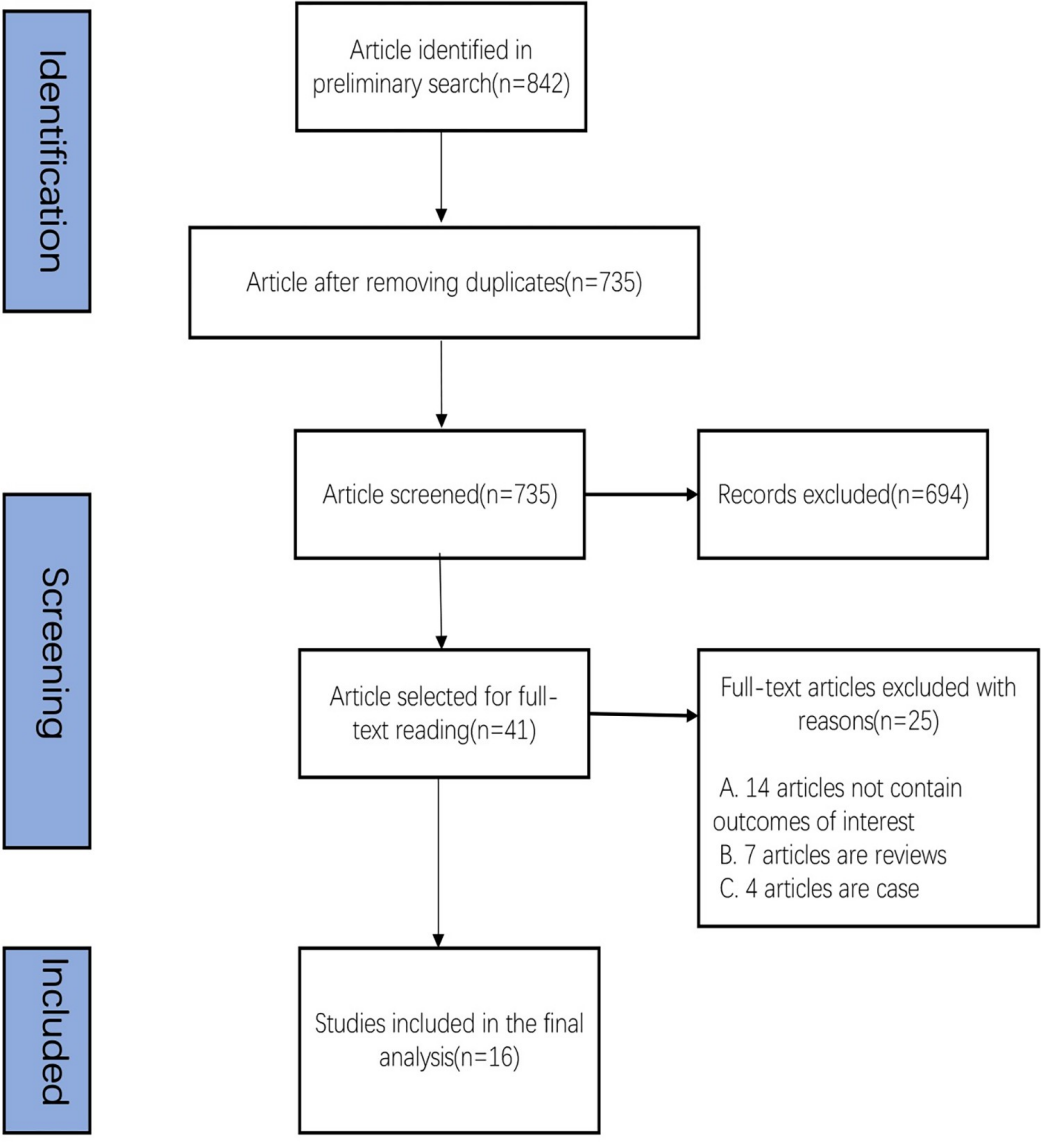
Literature search and review flowchart for study selection.

We initially identified 842 records and included 16 studies involving 3676 participants in the meta-analysis. The flow diagram of the selected studies is presented in Fig 1. These studies were published between 2013 and 2022. The baseline characteristics of the included studies are provided in Table 1. Of these studies, six were from China, three were from Korea, one each was from United Kingdom, Australia, Brazil, Italy, Chile, America, and Greece. All included studies were randomized controlled trials, and their full texts were published. The participants were mainly ACS patients who underwent PCI.

**Table 1:** Characteristics of the included studies.

Study	Published year	Country	Population	Sample size and intervention	Age(y) (corresponding to intervention)	Duration	Presented data
James Xu	2022	Australia	Patients with NSTEMI	60, ticagrelor (180mg loading dose, 90mg twice daily), 58, clopidogrel (600 mg loading dose, 75 mg once daily)	58.5±11.7 59.8±12.1	—	IMR, CFR, TIMI
Kyungil Park	2019	Korea	patients with ACS	60, ticagrelor (180mg loading dose, 90 mg twice daily), 60, clopidogrel (600mg loading dose, 75mg once daily)	56.9±11.4 58.5 ± 9.9	6 months	IMR, CFR
Sang-Don Park	2016	Korea	Patients with STEMI	38, ticagrelor (180mg loading dose, 90mg twice daily), 38, clopidogrel (600mg loading dose, 75mg once daily)	61.5±11.9 56.3±13.3	—	IMR, CFR
Woong Gil Choi	2014	Korea	patients with ACS	12, ticagrelor (180mg loading dose, 90mg twice daily); 12, clopidogrel (300mg loading dose, 75mg once daily)	—	—	IMR, CFR
Bangming Cao	2019	China	Patients with STEMI	49, ticagrelor (180 mg loading dose, 90 mg twice daily), 48, clopidogrel (600 mg loading dose, 75 mg once daily)	61.59±11.22 62.79 ± 11.37	7 days	TIMI, cTFC
José Luis Winter	2014	Chile	Patients with STEMI	34, ticagrelor (180 mg loading dose, 90 mg twice daily), 36, clopidogrel (600 mg loading dose, 75 mg once daily)	55.1±8.3 62.1±10.5	—	MBG, TIMI, cTFC
José R Mont’Alverne-Filho	2016	Brazil	Patients with STEMI	46, ticagrelor (180 mg loading dose, 90 mg twice daily), 44, clopidogrel (600 mg loading dose, 75 mg once daily)	58(IQR 52–69) 58(IQR 51.3–64)	12 hours	MBG, TIMI
K Zhu	2015	China	Patients with AMI	37, ticagrelor (180 mg loading dose, 90 mg twice daily), 37, clopidogrel (600 mg loading dose, 75 mg once daily)	59.5±4.63 60.2 ± 4.23	1 month	TIMI
Luca Di Vito 2016	2016	Italy	Patients with STEMI	39, ticagrelor (180 mg loading dose, 90 mg twice daily), 44, clopidogrel (600 mg loading dose, 75 mg once daily)	63(IQR58–69) 66(IQR 54–80)	—	MBG, TIMI, cTFC
Xuechao Wang	2019	China	Patients with STEMI	150, ticagrelor (180 mg loading dose, 90 mg twice daily), 148, clopidogrel (600 mg loading dose, 75 mg once daily)	102.87±20.82 98.76 ± 22.16	1 month	MBG, TIMI, cTFC
Y. LIU	2019	China	Patients with ACS	108, ticagrelor (180 mg loading dose, 90 mg twice daily), 100, clopidogrel (600 mg loading dose, 75 mg once daily)	55.1±8.3 62.1 ± 10.5	1 month	TIMI
Vijay Kunadian	2013	United Kingdom	Patients with ACS	1053, ticagrelor (180 mg loading dose, 90 mg twice daily), 1015, clopidogrel (600 mg loading dose, 75 mg once daily)	61.0±10.9 61.1 ± 10.8	6 months	TIMI, cTFC
Eun Kyong Kin	2017	America	Patients with STEMI	45, ticagrelor (180 mg loading dose, 90 mg twice daily), 50, clopidogrel (600 mg loading dose, 75 mg daily)	—	—	TIMI
Stylianos petousis	2022	Greece	Patients with STEMI	21 ticagrelor (180 mg loading dose, 90 mg twice daily), 21, clopidogrel (300mg loading dose, 75 mg once daily)	53.71±9.33 57.9±7.04	—	TIMI
Yang Liu	2017	China	Patients with STEMI	86, ticagrelor (180 mg loading dose, 90 mg twice daily), 87, clopidogel (300 mg loading dose, 75 mg once daily)	59.1±9.8 57.5±7.9	—	TIMI
WenHuaLi	2018	China	Patients with STEMI	20, ticagrelor (180 mg loading dose, 90 mg twice daily), 20, clopidogrel (600 mg loading dose, 75 mg once daily)	58.54±16.48 59.45±10.98	—	,cTFC

ACS: Acute coronary syndrome, STEMI:ST-segment elevation myocardial infarction, IMR: Index of microcirculatory resistance, CFR: Coronary flow reserve, MBG: Myocardial blush grade, TIMI: Thrombolysis in myocardial infarction, cTFC: Corrected thrombolysis in myocardial infarction frame count.

Of the 16 included primary studies, four studies reported differences in IMR, CFR and MBG after ticagrelor administration, five studies reported differences in cTFC, and twelve studies reported differences in TIMI.

**Fig 2:**
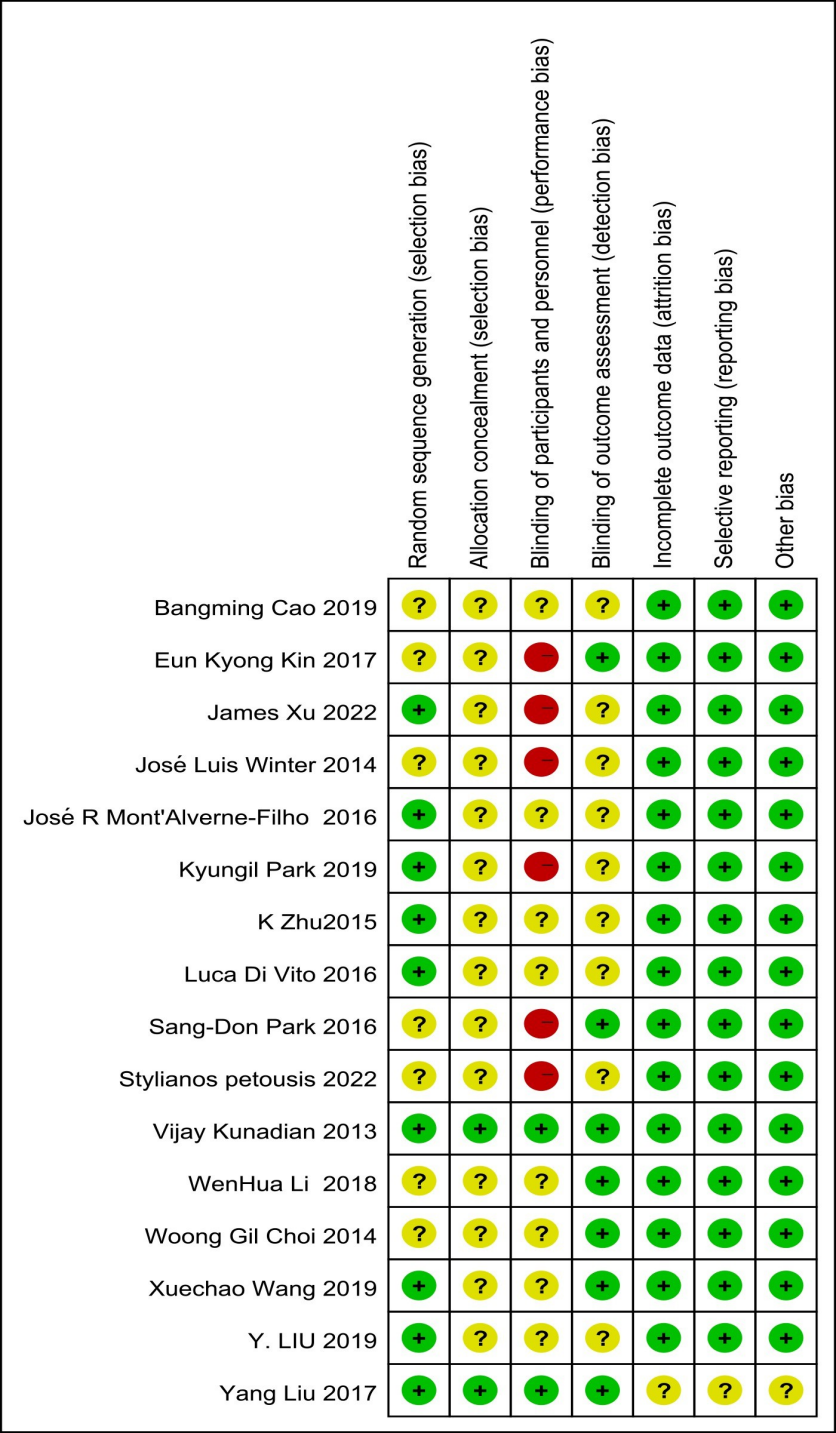
Risk of bias assessment of the included studies.

The risk of bias is shown in Fig 2. The low level of reported blinding for participants and investigators was the key limitation. Eight studies were at unclear risk of bias in allocation concealment, blinding of participants and personnel and outcome assessment. In six of the sixteen studies, there was a high risk of bias for implementation and measurement in terms of blinding of participants and personnel. There is no evidence of publication bias through visualization of funnel plots and the results of Egger’s and Begg’s tests. According to the GRADE tool, the quality of evidence for the primary outcome was moderate or low.

### Primary outcome

#### Effect of ticagrelor on IMR

**Fig 3:**
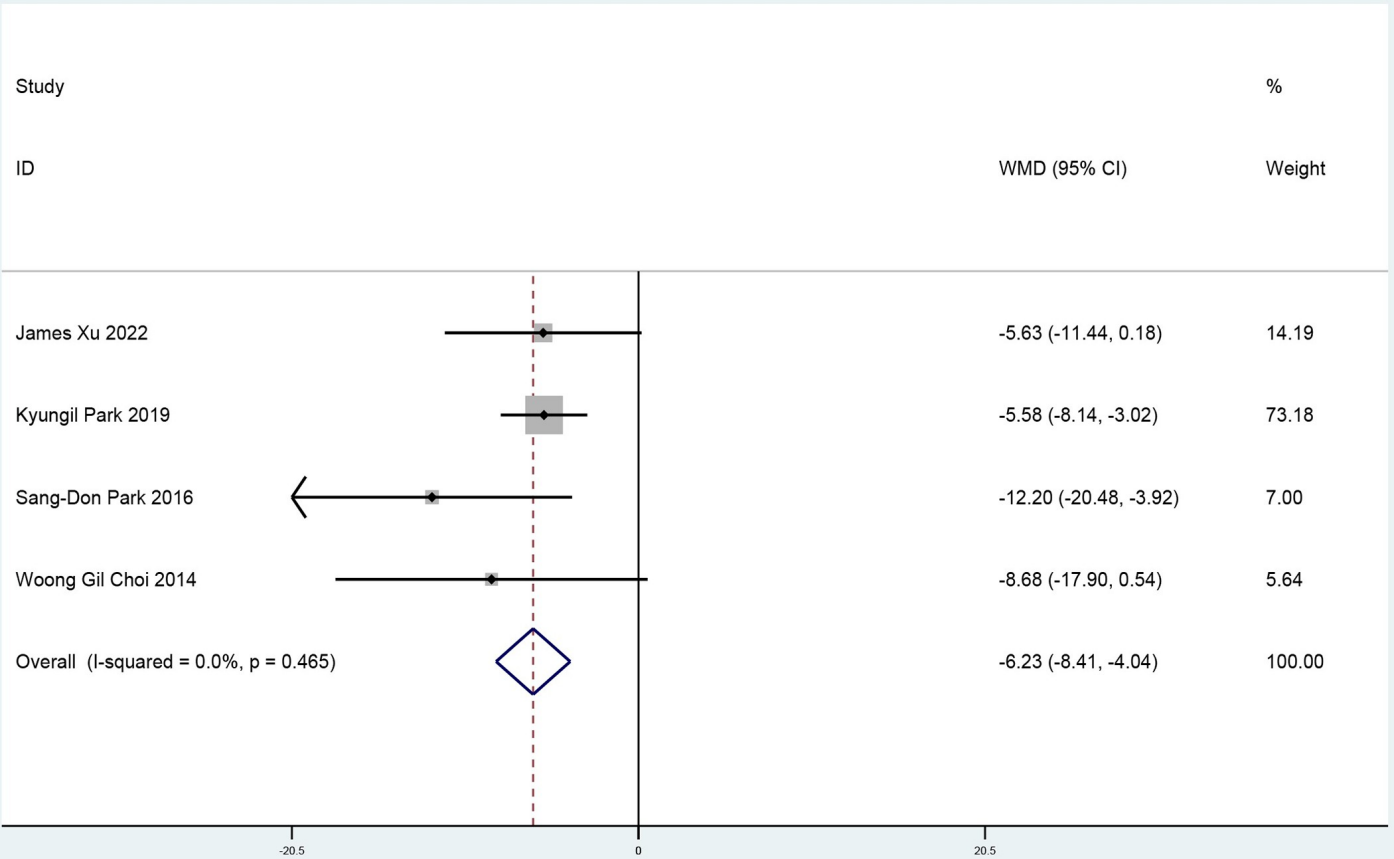
A pooled estimate of the ticagrelor effect on the index of microcirculatory resistance.

Four studies assessed IMR (170 participants in the ticagrelor group and 168 participants in the control group) and showed that ticagrelor significantly lowered IMR (weighted mean difference -6.23, 95% confidence interval: -8.41 to -4.04, p<0.001; Fig 3) compared with the control group, with low heterogeneity (I^2^ = 0%, P = 0.46). We observed no evidence of publication bias when we used Egger’s test and Begg’s test (S1 and S2 Figs). According to the GRADE assessment, there was moderate-quality evidence for the effect of IMR.

#### Effect of ticagrelor on CFR

**Fig 4:**
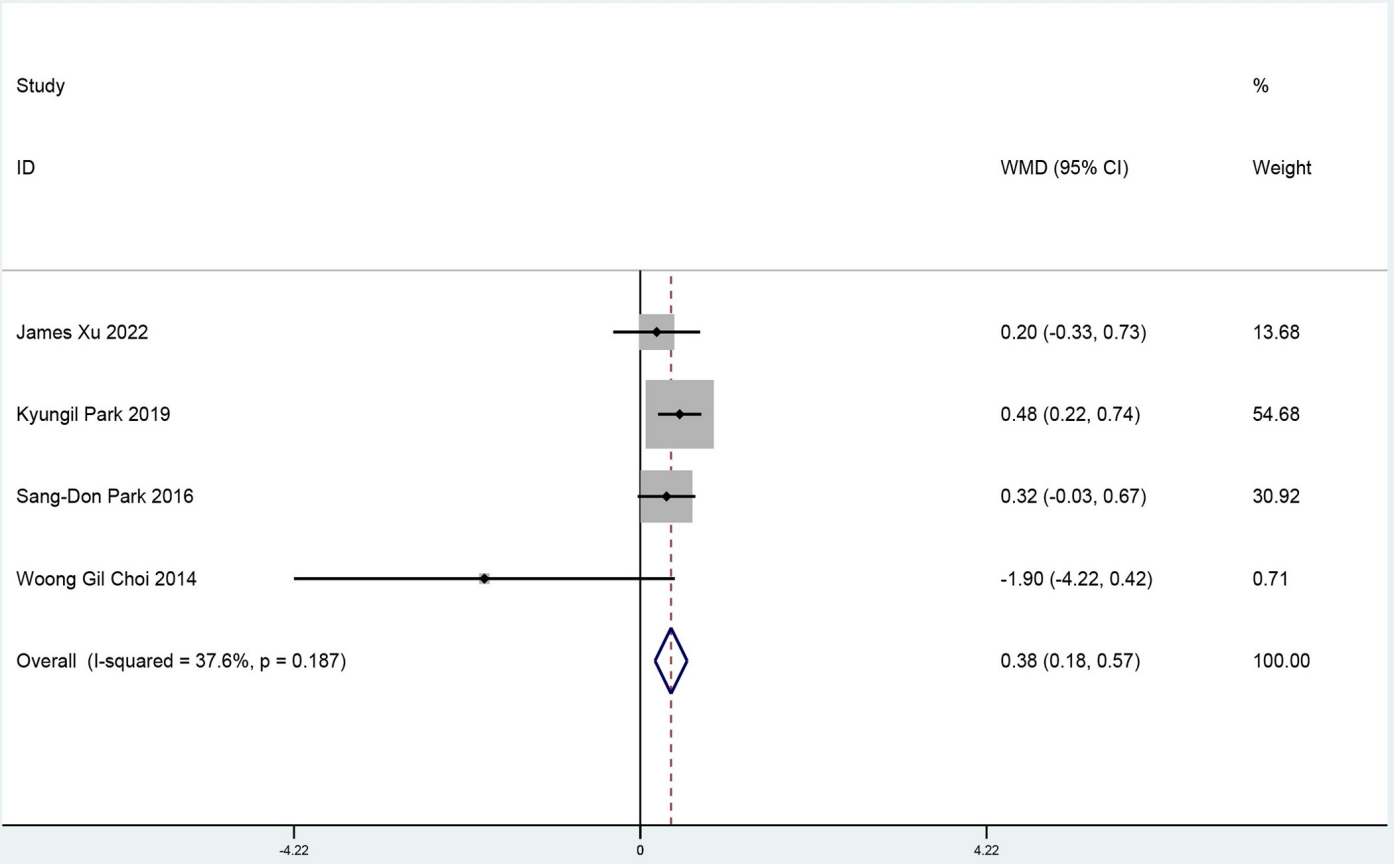
A pooled estimate of the ticagrelor effect on coronary flow reserves.

Four studies analyzed CFR with 338 participants (170 participants in the ticagrelor group and 168 participants in the control group). Compared with clopidogrel, ticagrelor increased the CFR level (weighted mean difference 0.38; 95% confidence interval 0.18 to 0.57, p<0.001; Fig 4). Test of heterogeneity yielded I^2^ = 37.6%, indicating low heterogeneity between two groups. Egger’s and Begg’s tests showed no evidence of publication bias (S3 and S4 Figs). The GRADE assessment showed that there was low-quality evidence for the effect of CFR.

### Secondary outcome

#### Effect of ticagrelor on MBG

**Fig 5:**
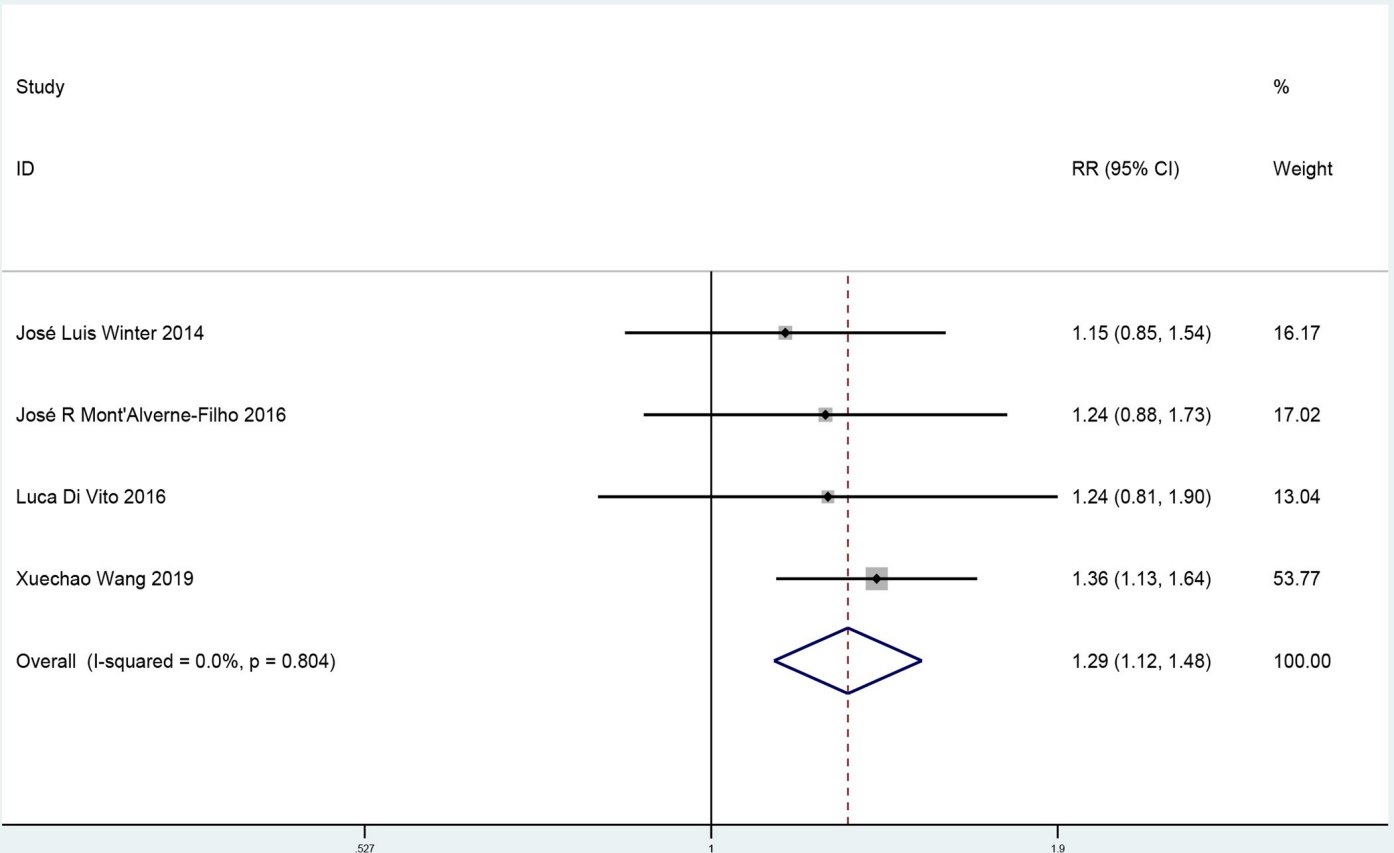
A pooled estimate of the effect of ticagrelor on myocardial blush grade.

Four studies analyzed MBG with 541 participants (269 participants in the ticagrelor group and 272 participants in the control group). Compared with patients in the control group, patients in the ticagrelor group showed a 29% increased level of MBG (risk ratio 1.29, 95% confidence interval 1.12 to 1.48; Fig 5). No publication bias was found (Egger’s test, *p * =  1.00, S5 and S6 Figs).

#### Effect of ticagrelor on TIMI myocardial perfusion grade III

**Fig 6:**
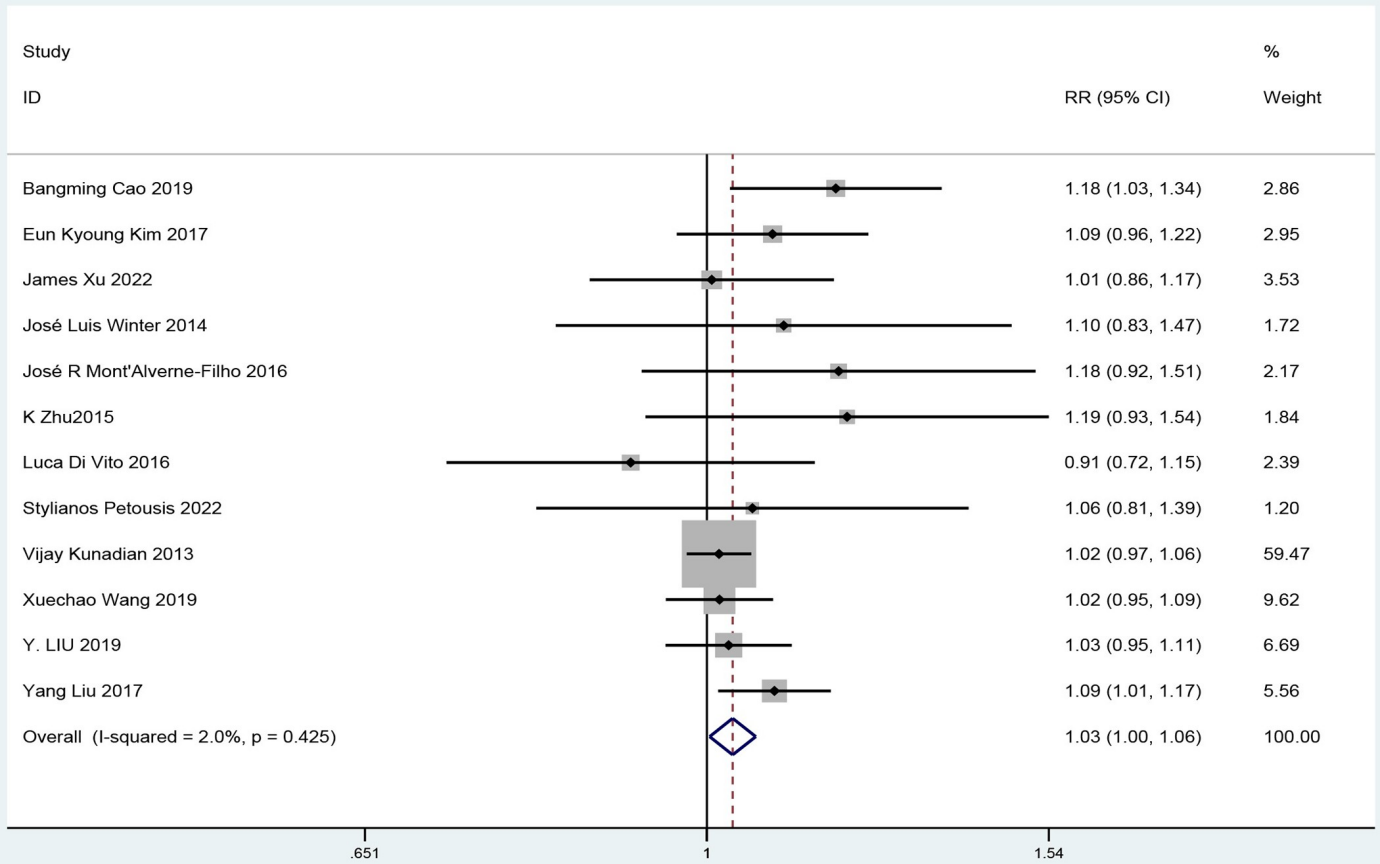
A pooled estimate of the effect of ticagrelor on thrombolysis in myocardial infarction.

The efficacy of ticagrelor administration on TIMI grade III was investigated in twelve studies (1728 participants in the ticagrelor group and 1688 participants in the control group). Compared with patients in the clopidogrel group, patients in the ticagrelor group showed a 3% increase in TIMI blood flow III risk ratio 1.03, 95% confidence interval 1.00 to 1.06; Fig 6). Heterogeneity was very low (I^2^ = 2%, P = 0.42). We found no evidence of asymmetry in the funnel plot (S7 Fig).

#### Effect of ticagrelor on cTFC

**Fig 7:**
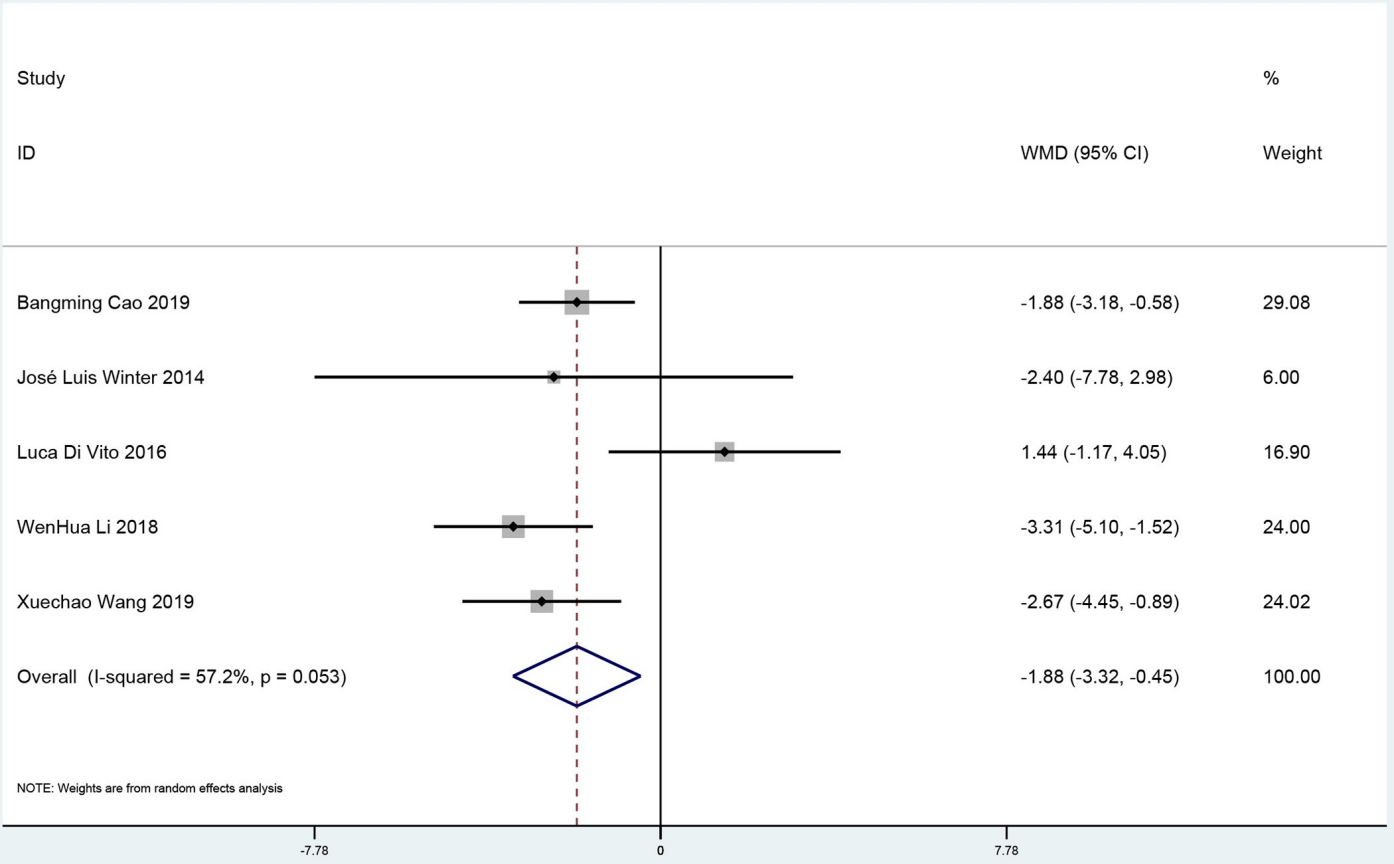
A pooled estimate of ticagrelor effect on the corrected thrombolysis in myocardial infarction frame count.

Five studies analyzed cTFC with 588 participants (292 participants in the ticagrelor group and 296 participants in the control group). Compared with clopidogrel, it concluded that the patients who were treated with ticagrelor had lower level of cTFC after PCI (weighted mean deviation -1.88, 95% confidence interval -3.32 to -0.45; Fig 7) in the random-effect model. Neither Egger’s test nor Begg’s test showed evidence of publication bias for the effect of ticagrelor on MBG (S8 and S9 Figs). According to the GRADE assessment, there was low-quality evidence for the effects of ticagrelor on MBG, TIMI grade III, and cTFC. (S2 Table).

## Discussion

P2Y12 receptor antagonists, such as thienopyridines (e.g., clopidogrel) and ticagrelor, are commonly used antiplatelet medications that inhibit platelet activation and aggregation, thus preventing arterial thrombus formation. However, emerging research suggests that their effects and clinical benefits may extend beyond platelet inhibition [27]. Thienopyridines are prodrugs of which the active metabolite, after bioactivation by CYP P450 liver enzymes, irreversibly bind to the P2Y12 receptor. Ticagrelor also inhibits cellular adenosine uptake through ENT1. These differences in binding characteristics and additional mode of action provide distinct P2Y12 inhibition profiles and contribute to the divergent clinical effects observed between thienopyridines and ticagrelor [28]. Clinical studies have also indicated that ACS patients treated with ticagrelor have shown improved peripheral vascular endothelial function compared to those on clopidogrel [28]. In this meta-analysis of 16 randomized controlled trials with a total of 3676 participants, the use of ticagrelor was significantly associated with the improvement of coronary microvascular function. The findings indicate that ticagrelor significantly increased CFR, MBG, and TIMI and significantly reduced IMR and cTFC compared with clopidogrel. Many large clinical randomized controlled trials shown that ticagrelor reduces the incidence of CMVD of patients with ACS. This is consistent with the results of our meta-analysis.

### Potential underlying mechanisms

Many clinical trials have shown that ticagrelor is more effective than clopidogrel in terms of improving the prognoses of patients with ACS who receive PCI [1,29,30]. In the Platelet Inhibition and Patient Outcomes (PLATO) study, treatment with ticagrelor compared to clopidogrel in patients with ACS significantly reduced mortality from vascular causes, myocardial infarction, or stroke [30]. The precise mechanisms of how ticagrelor plays a key role in the development of coronary microcirculation in patients with ACS post-PCI remain unknown. Several possible mechanisms have been suggested. One of the possibilities is the unique antiplatelet effect of ticagrelor. Compared with clopidogrel, ticagrelor offers a rapid onset of action, enabling the exertion of antiplatelet effects within a brief timeframe. Additionally, the action of ticagrelor is reversible, and its antiplatelet effects are rapidly reversed upon discontinuation. This characteristic makes it easier to control platelet function in situations where surgical procedures or an increased risk of bleeding are involved. In comparison, clopidogrel requires hepatic metabolism to generate its active metabolites. As a result, it has a longer onset time and requires some time to reach its maximum antiplatelet effects. Overall, more potent and consistent antiplatelet effects of ticagrelor play a relevant role in protecting against thrombus microembolization and improving myocardial perfusion [8].

Another mechanism is related to an interaction with adenosine metabolism, which might contribute to its effect of improving microvascular function [31]. Adenosine, as a naturally occurring endogenous purine nucleotide [32], is known to improve the recovery of cardiac function after ischemia by regulating myocardial and coronary circulatory functions [33]. Inflammatory pathways and leucocyte infiltration after PCI can cause disturbance of coronary microcirculation [34]. Adenosine can modulate the inflammatory responses to various stressful conditions by inhibiting neutrophil trafficking and inhibiting the production of reactive oxygen species and inflammatory mediators [35]. In addition, ticagrelor can promote the adenosine-induced release of NO, which can reduce ischemia/reperfusion injury [36]. Moreover, adenosine may prevent microvascular dysfunction and reduce endothelial cell injury [37].

Current research has indicated that adenosine is rapidly taken up by cells through sodium-independent equilibrative nucleoside transporters (ENT1/2) and sodium-dependent concentrative nucleoside transporters (CNT2/3) [38,39]. Ticagrelor can inhibit adenosine degradation, which is mediated by inhibiting adenosine uptake into erythrocytes [38]. A recent study in patients with coronary heart disease revealed that patients receiving ticagrelor had significantly higher adenosine plasma concentration (APC) than patients receiving clopidogrel [39]. A study indicated that a 180-mg loading dose of ticagrelor has microvascular protective potential in STEMI patients, which might be associated with its adenosine-dependent mechanism [40].

Interestingly, during our search process, we found a few studies that suggest a more pronounced improvement in coronary microcirculation after PCI with prasugrel compared to ticagrelor [41]. Compared with clopidogrel, ticagrelor has greater antiplatelet effects [42], but ticagrelor and prasugrel have comparable effects [43]. Moreover, the possible benefit of prasugrel, in comparison with ticagrelor, may be related to improved endothelial function, which may result in similar effects on microvascular function between ticagrelor and prasugrel [44]. However, only a few studies compared the effect of ticagrelor with the effects of prasugrel, which substantially reduced the statistical power and limited our results to the comparison of efficacy between ticagrelor and clopidogrel. In the future, we expect the quality of evidence to improve and more high-quality studies to emerge.

### Strengths and limitations of the study

This meta-analysis assessed the efficacy of ticagrelor on coronary microcirculation in patients with ACS. This systematic review and meta-analysis have several methodological strengths. We followed the principle of the Cochrane Collaboration and the PRISMA statement. Moreover, our review used robust methods, including strict quality assessment by the GRADE approach.

Both invasive and noninvasive techniques have relative advantages and pitfalls. In order to comprehensively evaluate coronary microcirculation function, our review used various effect sizes to comprehensively evaluate the change in microvascular function and obtain a complete understanding of the efficiency of ticagrelor. These methods include measurement of coronary blood flow velocity, assessment of myocardial blood flow, and calculation of the microcirculatory resistance index. According to the effectiveness and reproducibility of these techniques, we divided these methods into primary and secondary outcomes.

Our study had several limitations. First, as with all meta-analyses, our study was limited by the quality of the included studies. The studies included in our meta-analysis were regarded as having either a moderate risk of bias, mainly because of serious bias due to the low level of reported blinding for participants and investigators. Moreover, the GRADE approach is the main source of low-quality evidence of study outcomes due to the limitations of the study design and potential confounding bias without sufficient adjustment for confounders. Second, there are various differences in study designs and different subtypes of ACS, although the low heterogeneity partially proved that our results were statistically reliable. Thirdly, most measurements of coronary microvascular function in experimental studies are conducted within six months after the loading dose of P2Y12 inhibitors. Therefore, future studies will need to maintain the dose for a longer duration to investigate the long-term effects of ticagrelor on coronary microcirculation. However multiple trials suggest that the achieved antiplatelet effect at the time of reperfusion is of more importance than later during the follow-up [45]. In order to incorporate a multiple treatment network model for a more precise assessment of ticagrelor’s efficacy, we expect the quality of evidence to improve and more high-quality studies to emerge in the future.

## Conclusion

Our systematic review and meta-analysis showed that ticagrelor treatment can ameliorate coronary microvascular function compared to clopidogrel treatment. The unique function of ticagrelor may result from its stronger and quicker antiplatelet action and interaction with adenosine.

## Supporting information

S1 Checklist PRISMA checklist. (DOCX)

S1 Fig Publication bias assessment by used Begg test for IMR. (TIF)

S2 Fig Publication bias assessment by used Egger’s test for IMR. (TIF)

S3 Fig Publication bias assessment by used Begg test for CFR. (TIF)

S4 Fig Publication bias assessment by used Egger’s test for CFR. (TIF)

S5 Fig Publication bias assessment by used Begg test for MBG. (TIF)

S6 Fig Publication bias assessment by used Egger’s test for MBG. (TIF)

S7 Fig The funnel plot of TIMI myocardial perfusion grade III. (TIF)

S8 Fig Publication bias assessment by used Begg test for cTFC. (TIF)

S9 Fig Publication bias assessment by used Egger’s test for cTFC. (TIF)

S1 Table Search strategy. (DOCX)

S2 Table Quality assessment of included studies according to the GRADE framework. (DOCX)

S1 File Included studies. (DOCX)
