# Supplementary material for: The effect of ticagrelor on coronary microvascular function after PCI in patients with ACS compared to clopidogrel: A systematic review and meta-analysis
Source: PLoS One. 2023 Aug 29;18(8):e0289243. doi: 10.1371/journal.pone.0289243 (PMC10464986; doi:10.1371/journal.pone.0289243)
Supplement: S1 Checklist — (DOCX) [file pone.0289243.s001.docx]

| **Section and Topic** | **Item #** | **Checklist item** | **Location where item is reported** |
| --- | --- | --- | --- |
| **TITLE** | | |  |
| Title | 1 | The report is Identified as a meta-analysis. | 1 |
| **ABSTRACT** | | |  |
| Abstract | 2 | The structured abstract included Objective, Research Design and Methods, Results and Conclusions. | 2 |
| **INTRODUCTION** | | |  |
| Rationale | 3 | Described in the introduction. | 3 |
| Objectives | 4 | Stated in the introduction. | 3 |
| **METHODS** | | |  |
| Eligibility criteria | 5 | Described in the methods. | 3 |
| Information sources | 6 | From inception to 12 May 2022, we comprehensively searched the PubMed, Cochrane Central Register of Controlled Trials (CENTRAL), and ClinicalTrials.gov databases for eligible RCTs. | 3 |
| Search strategy | 7 | Described in the methods. | 4 |
| Selection process | 8 | Described in the methods. | 4 |
| Data collection process | 9 | Described in the methods. | 4 |
| Data items | 10a | Described in the methods. | 4 |
|  | 10b | Described in the methods. | 4 |
| Study risk of bias assessment | 11 | Described in the methods. | 4 |
| Effect measures | 12 | Risk Ratio and Mean Difference used in the synthesis or presentation of results. | 4 |
| Synthesis methods | 13a | Described in the methods. | 4 |
|  | 13b | Described in the methods. | 4 |
|  | 13c | Described in the methods. | 4 |
|  | 13d | Described in the methods. | 4 |
|  | 13e | Described in the methods. | 4 |
|  | 13f | Described in the methods. | 4 |
| Reporting bias assessment | 14 | Described in the methods. | 4 |
| Certainty assessment | 15 | Described in the methods. | 4 |
| **RESULTS** | | |  |
| Study selection | 16a | Described in the results. | 5 |
|  | 16b | Described in the results. | 5 |
| Study characteristics | 17 | Described in the results. | 5 |
| Risk of bias in studies | 18 | Described in the results. | 5 |
| Results of individual studies | 19 | Described in the results. | 6 |
| Results of syntheses | 20a | Described in the results. | 6 |
|  | 20b | Described in the results. | 6 |
|  | 20c | Described in the results. | 6 |
|  | 20d | Described in the results. | 6 |
| Reporting biases | 21 | Described in the results. | 6 |
| Certainty of evidence | 22 | Described in the results. | 6 |
| **DISCUSSION** | | |  |
| Discussion | 23a | Described in the discussion. | 7 |
|  | 23b | Described in the discussion. | 9 |
|  | 23c | Described in the discussion. | 9 |
|  | 23d | Described in the discussion. | 8 |
| **OTHER INFORMATION** | | |  |
| Registration and protocol | 24a | The protocol for this review was registered with PROSPERO (CRD42022375073). | 3 |
|  | 24b | [PROSPERO (york.ac.uk)](https://www.crd.york.ac.uk/prospero/) | 3 |
|  | 24c | There is not have any amendments to information provided at registration or in the protocol. | 10 |
| Support | 25 | This research did not receive any specific grant from funding agencies in the public, commercial, or not-for-profit sectors. | 10 |
| Competing interests | 26 | The authors have no financial relationships with any organizations that might have an interest in the submitted work and no other relationships or activities that could appear to have influenced the submitted work. | 10 |
| Availability of data, code and other materials | 27 | The original contributions presented in the study are included in the article/Supplementary Material, and further inquiries can be directed to the corresponding authors. | 10 |

*From:*  Page MJ, McKenzie JE, Bossuyt PM, Boutron I, Hoffmann TC, Mulrow CD, et al. The PRISMA 2020 statement: an updated guideline for reporting systematic reviews. BMJ 2021;372:n71. doi: 10.1136/bmj.n71

For more information, visit: <http://www.prisma-statement.org/>
