# Supplementary figures and images for: The effect of ticagrelor on coronary microvascular function after PCI in patients with ACS compared to clopidogrel: A systematic review and meta-analysis
Source: PLoS One. 2023 Aug 29;18(8):e0289243. doi: 10.1371/journal.pone.0289243 (PMC10464986; doi:10.1371/journal.pone.0289243)

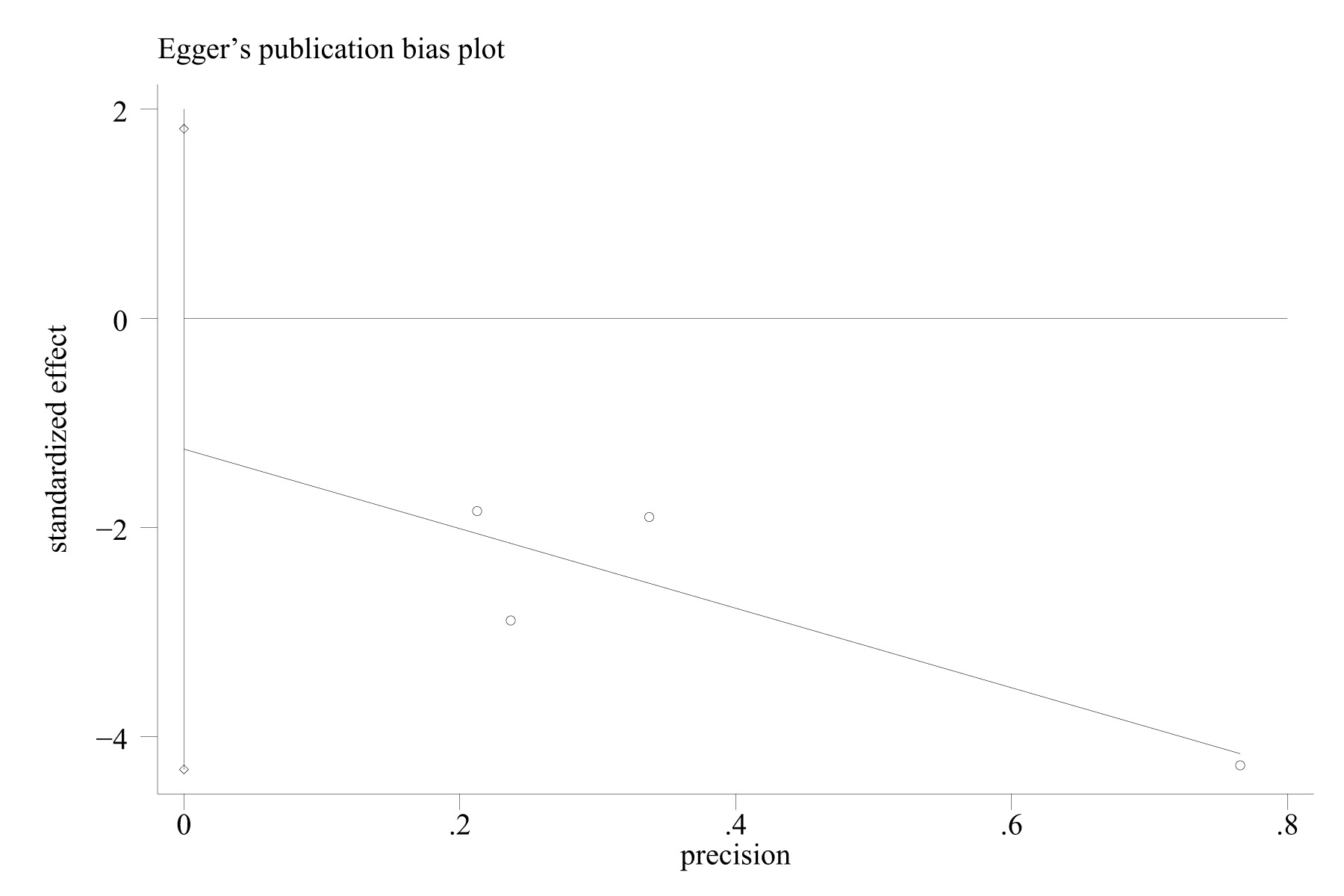

Supplement: S1 Fig — (TIF) [file pone.0289243.s002.tif]

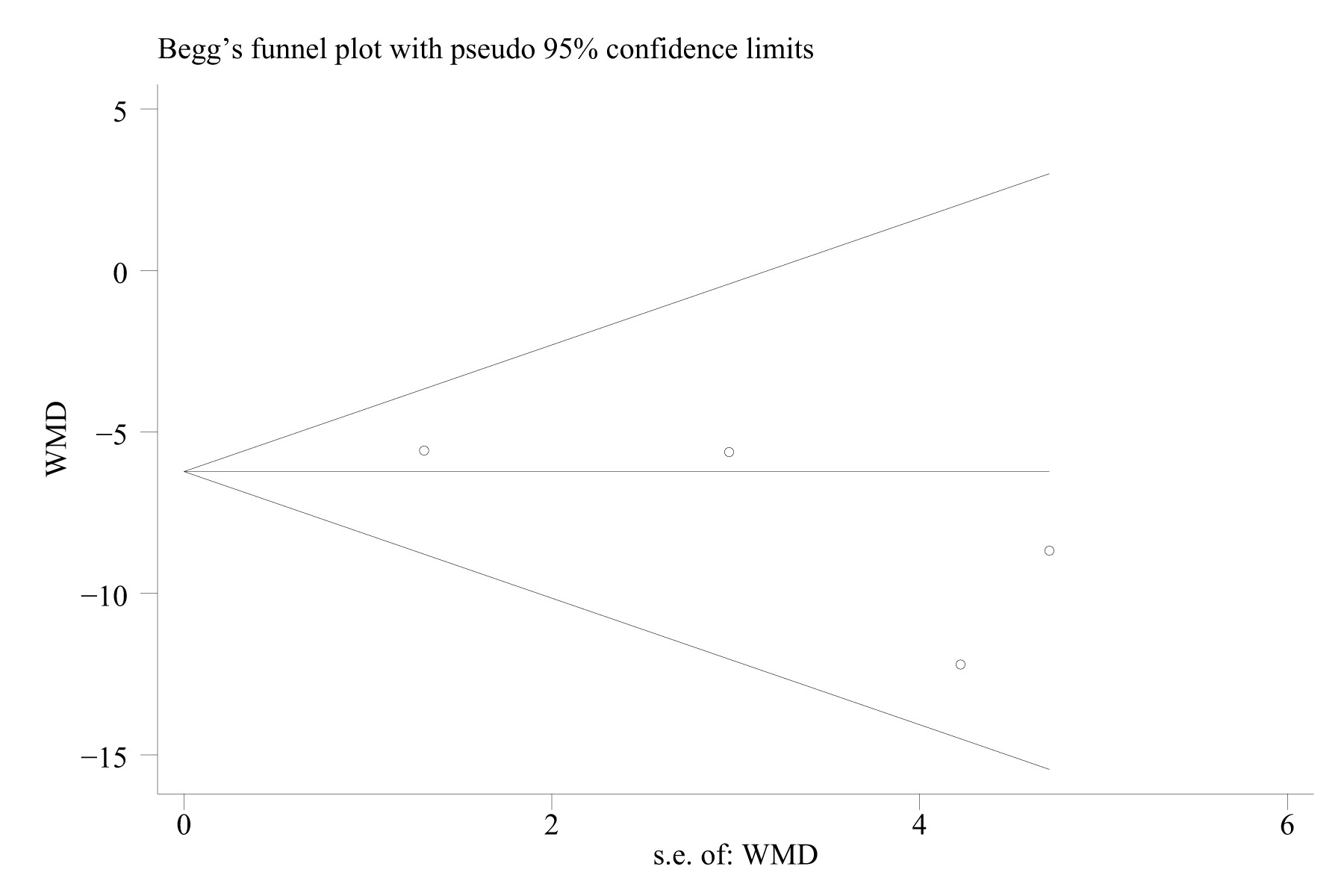

Supplement: S2 Fig — (TIF) [file pone.0289243.s003.tif]

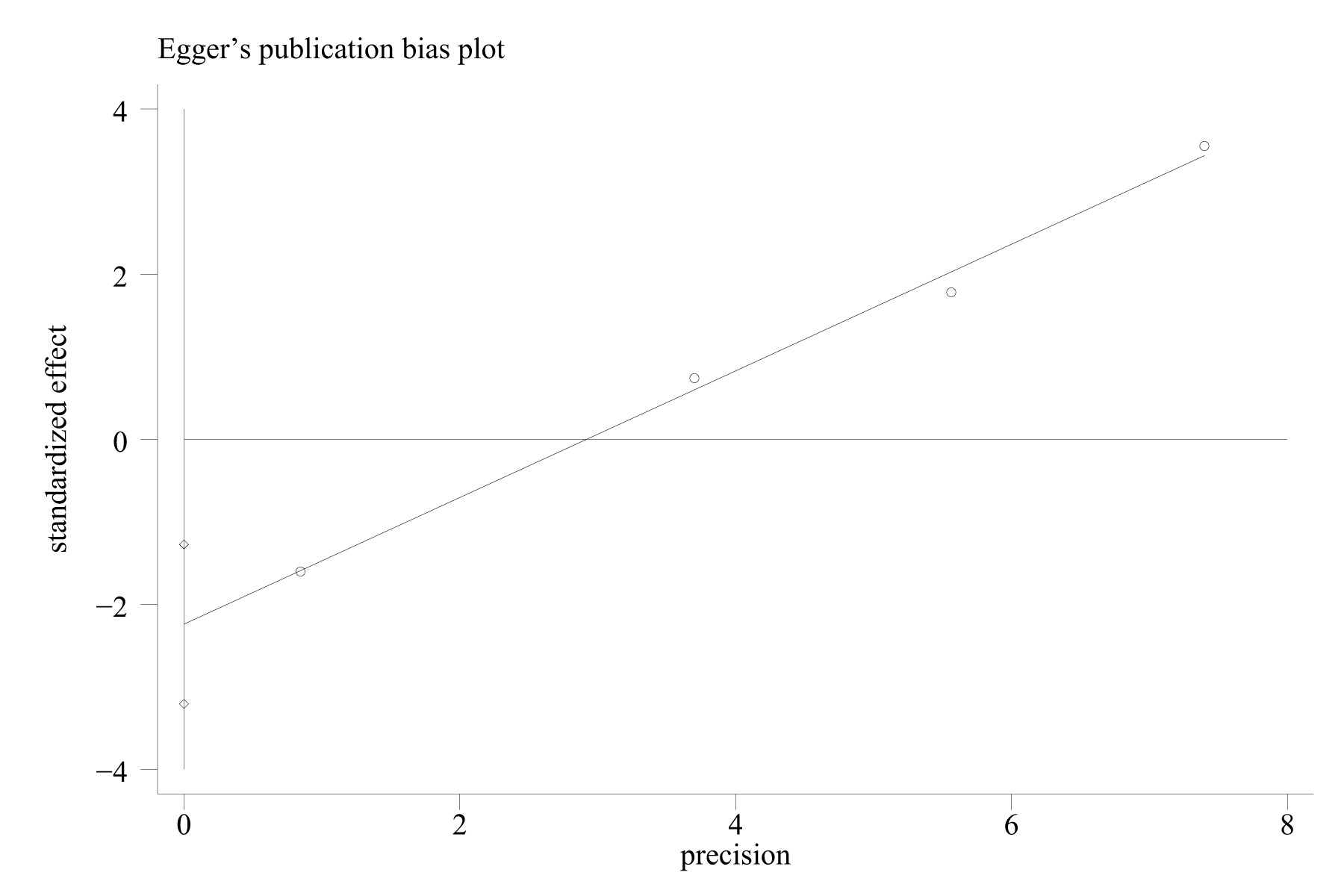

Supplement: S3 Fig — (TIF) [file pone.0289243.s004.tif]

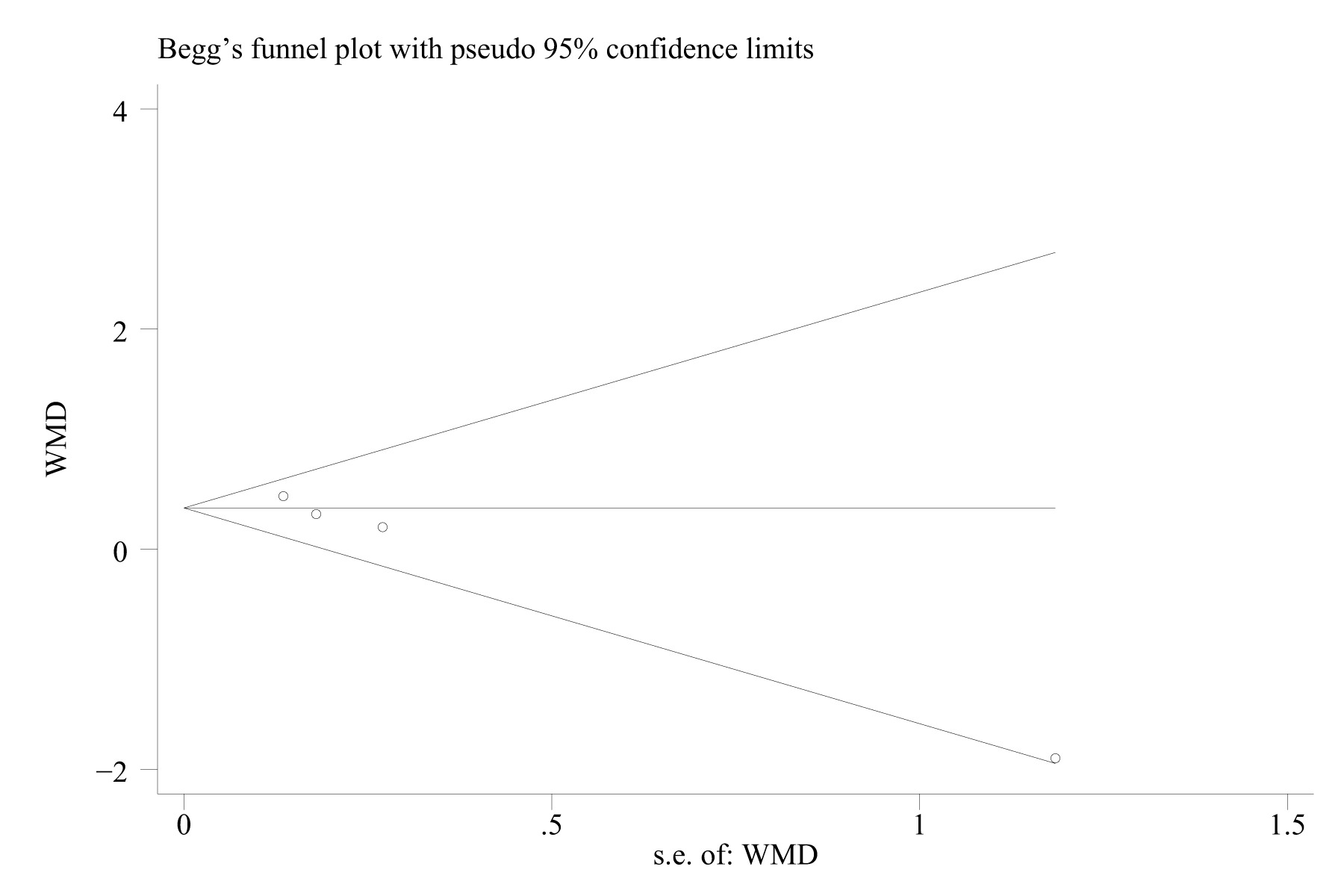

Supplement: S4 Fig — (TIF) [file pone.0289243.s005.tif]

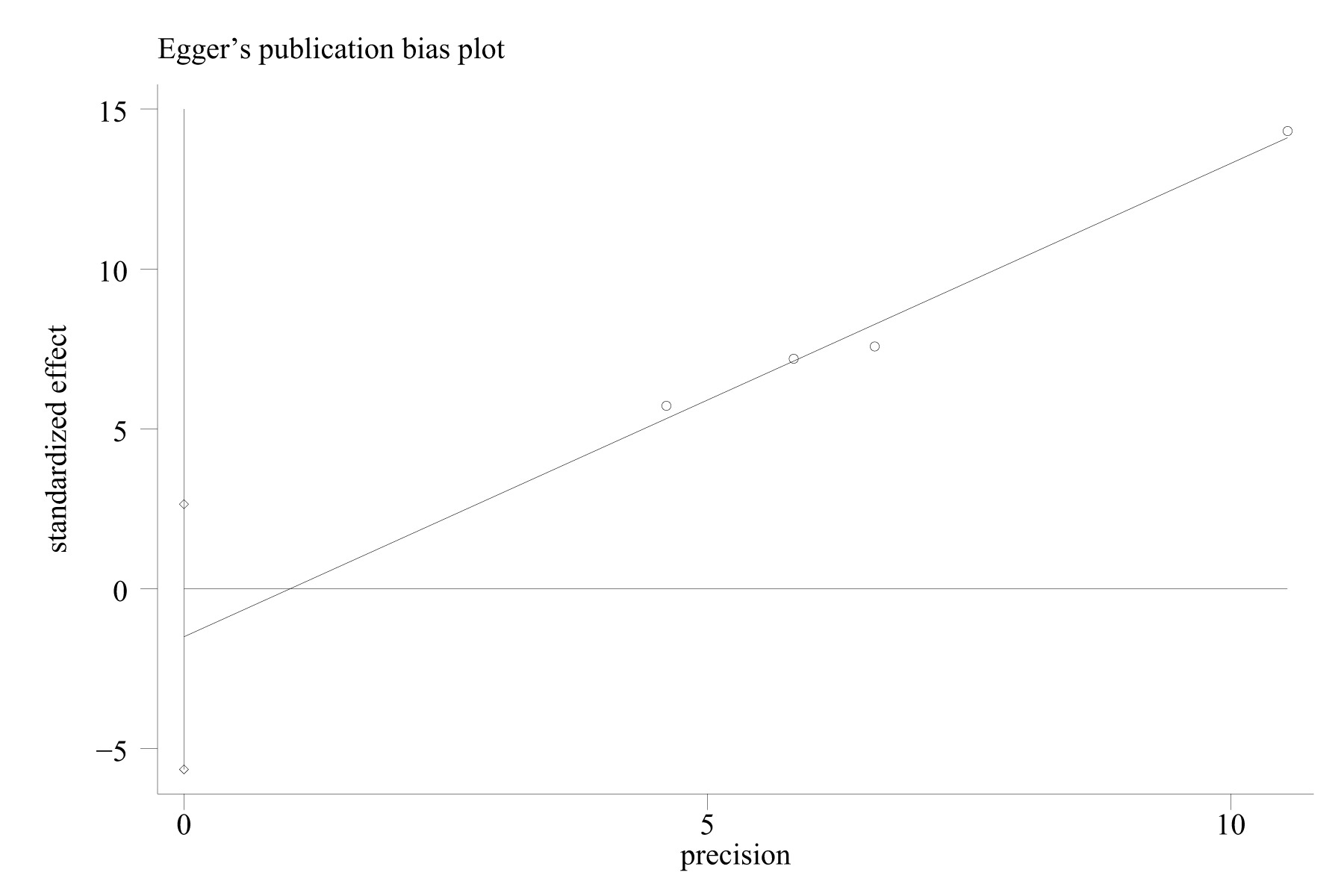

Supplement: S5 Fig — (TIF) [file pone.0289243.s006.tif]

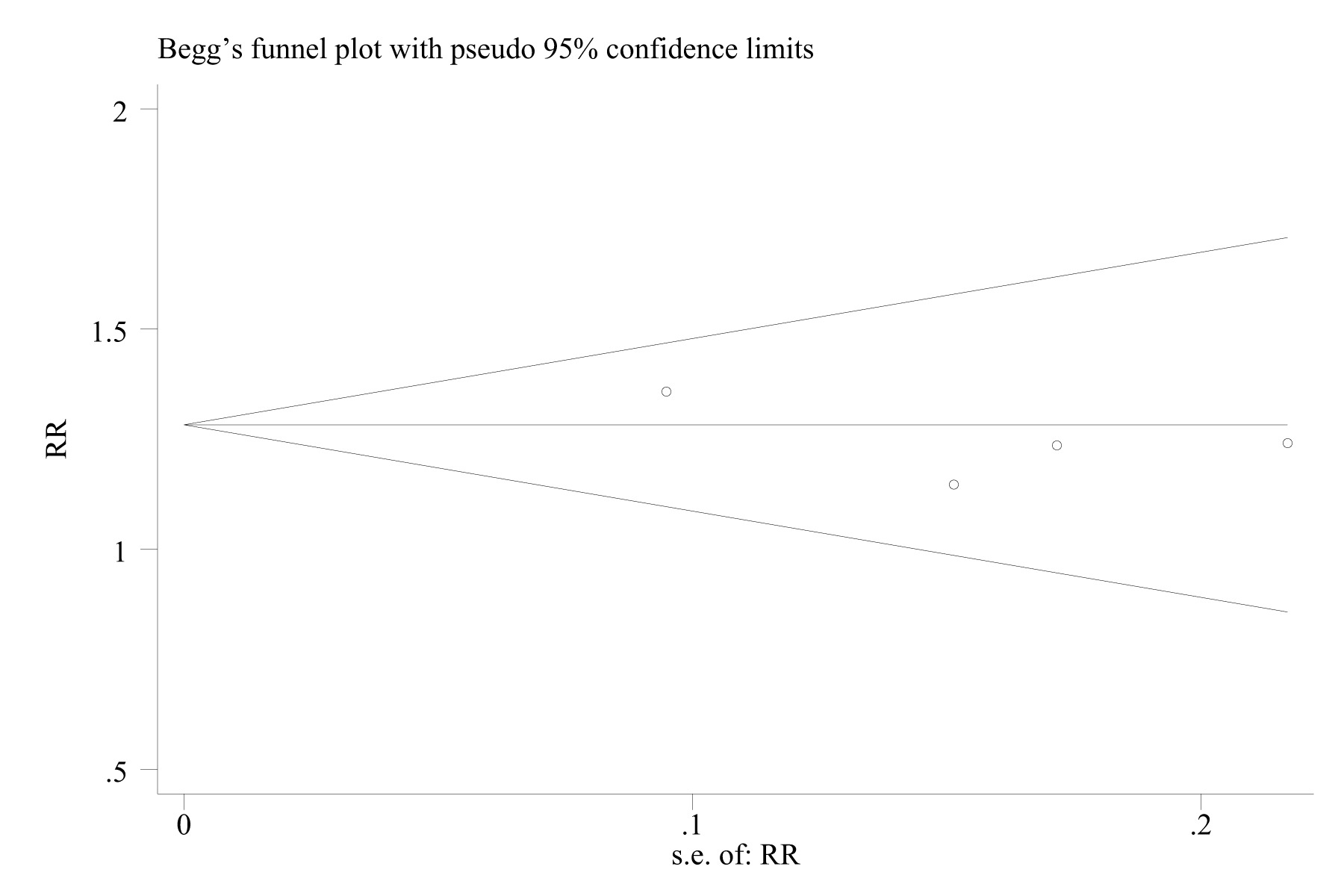

Supplement: S6 Fig — (TIF) [file pone.0289243.s007.tif]

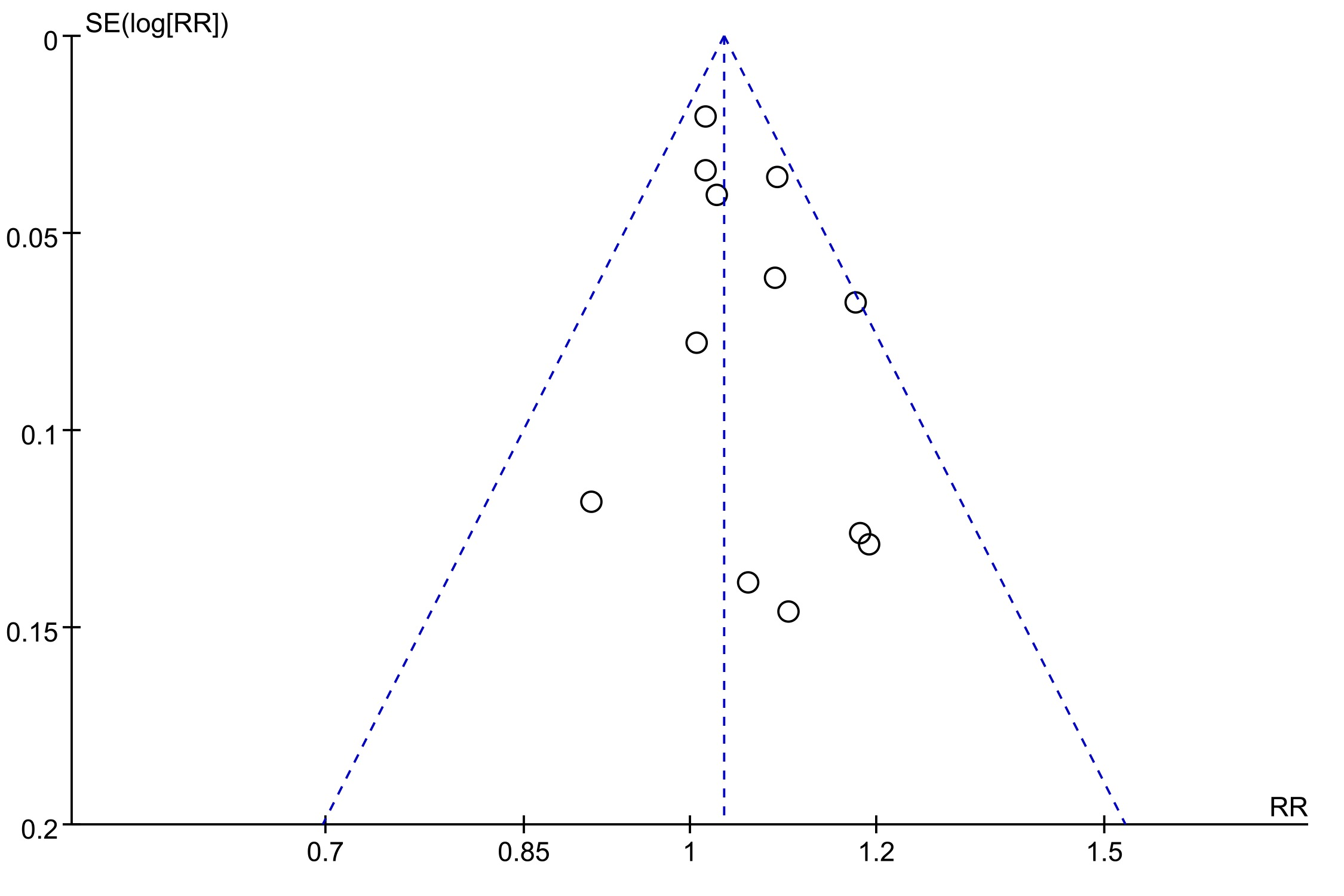

Supplement: S7 Fig — (TIF) [file pone.0289243.s008.tif]

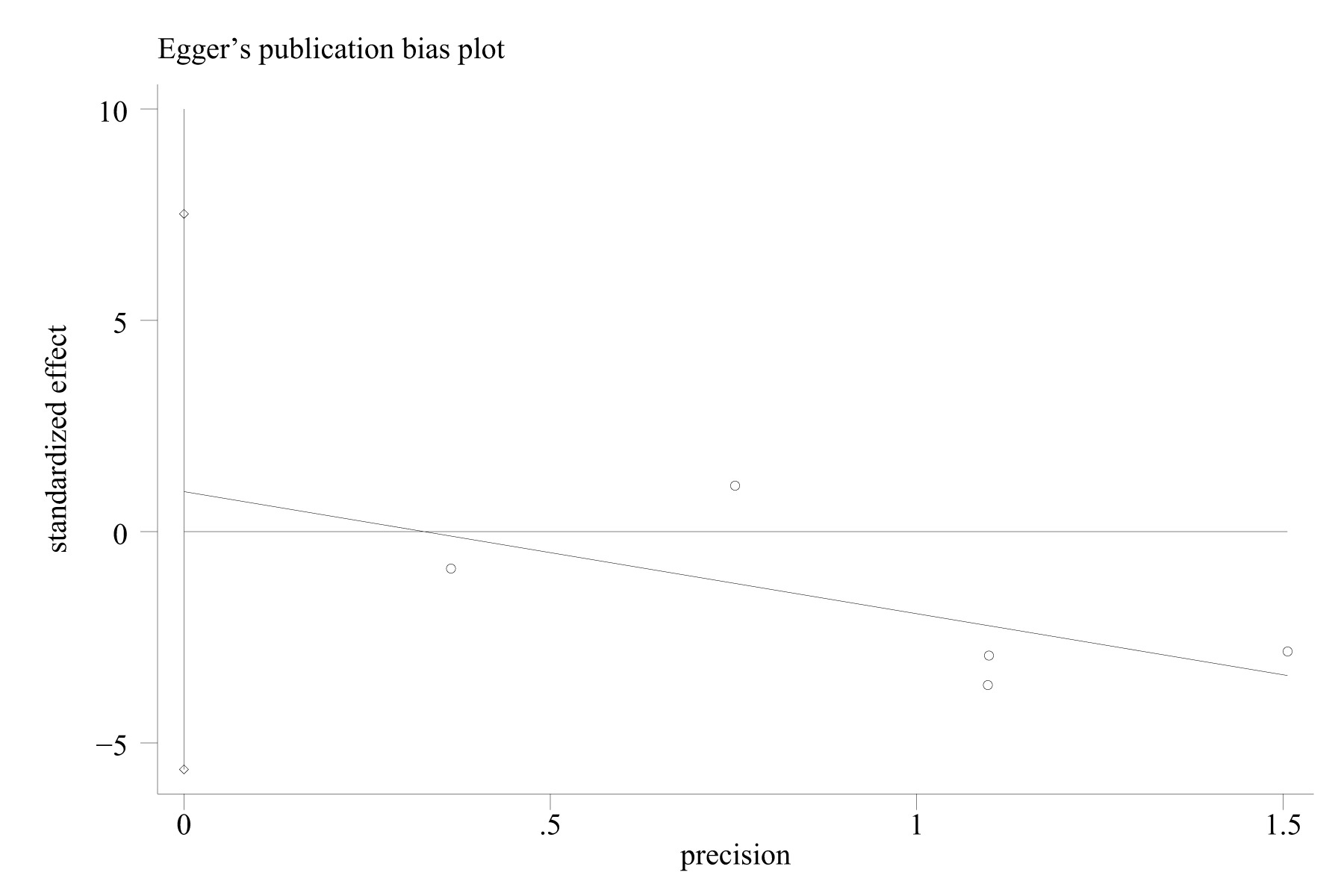

Supplement: S8 Fig — (TIF) [file pone.0289243.s009.tif]

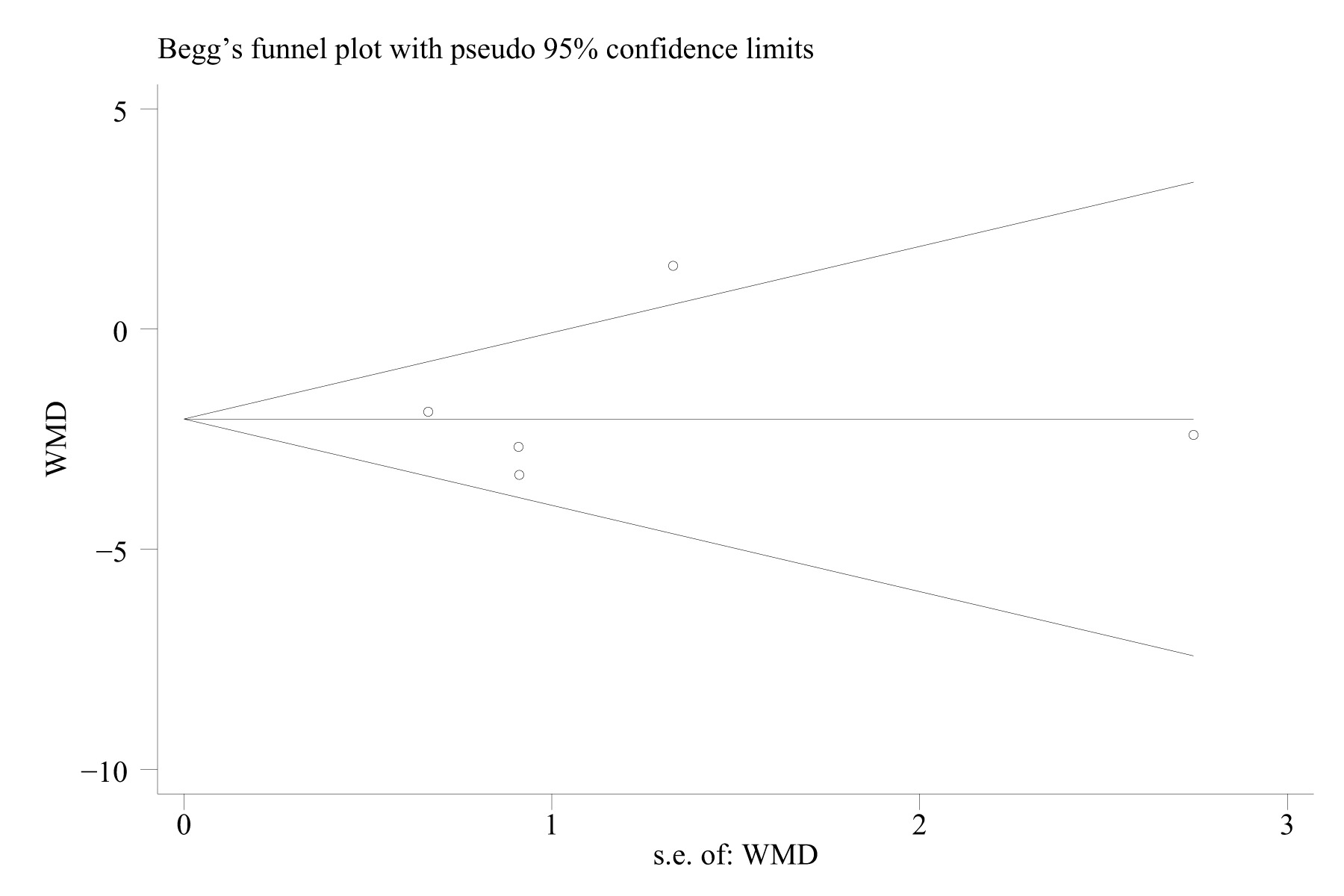

Supplement: S9 Fig — (TIF) [file pone.0289243.s010.tif]
