# Supplementary material for: The effect of ticagrelor on coronary microvascular function after PCI in patients with ACS compared to clopidogrel: A systematic review and meta-analysis
Source: PLoS One. 2023 Aug 29;18(8):e0289243. doi: 10.1371/journal.pone.0289243 (PMC10464986; doi:10.1371/journal.pone.0289243)
Supplement: S1 Table — (DOCX) [file pone.0289243.s011.docx]

| Database | Step | Search algorithm | Items  found |
| --- | --- | --- | --- |
| PubMed | 1 | ticagrelor | 3843 |
|  | 2 | Brilique | 3843 |
|  | 3 | AZD-6140 | 3847 |
|  | 4 | Brilinta | 3845 |
|  | 5 | Microvascular | 72220 |
|  | 6 | Microcirculation | 52657 |
|  | 7 | coronary artery | 313239 |
|  | 8 | coronary microvascular dysfunction | 6994 |
|  | 9 | CMVD | 145 |
|  | 10 | CMD | 3808 |
|  | 11 | randomized controlled trials | 800007 |
|  | 12 | RCT | 32639 |
|  | 13 | (ticagrelor) OR (Brilique) OR (AZD-6140) OR (Brilinta) OR (3-(7-((2- (3,4- Difluorophenyl) cyclopropyl) amino)-5-(propylthio) - 3H-(1-3)- triazolo(4,5-d) pyrimidin-3-yl) -5-(2-hydroxyethoxy) cyclopentane- 1,2-diol) | 3849 |
|  | 14 | (Microvascular) OR (Microcirculation) OR (coronary artery) OR (coronary microvascular dysfunction) OR (CMVD) OR (CMD) | 419757 |
|  | 15 | (Randomized controlled trials) OR (RCT) | 810845 |
|  | 16 | (((ticagrelor) OR (Brilique) OR (AZD-6140) OR (Brilinta) OR (3-(7-((2- (3,4-Difluorophenyl) cyclopropyl) amino) -5-(propylthio) -3H- (1-3)- triazolo(4,5-d) pyrimidin-3-yl) -5- (2-hydroxyethoxy) cyclopentane- 1,2-diol)) AND ((Microvascular) OR (Microcirculation) OR (coronary artery) OR (coronary microvascular dysfunction) OR (CMVD) OR (CMD))) AND ((randomized controlled trials) OR (RCT)) | 284 |
| Cochrane  Central Register of Controlled Trials (CENTRAL) | 1 | ticagrelor | 2182 |
|  | 2 | Brilique | 68 |
|  | 3 | AZD-6140 | 3 |
|  | 4 | Brilinta | 34 |
|  | 5 | Microvascular | 4373 |
|  | 6 | Microcirculation | 3002 |
|  | 7 | coronary artery | 37459 |
|  | 8 | coronary microvascular dysfunction | 358 |
|  | 9 | CMVD | 16 |
|  | 10 | CMD | 323 |
|  | 11 | Randomized controlled trials | 1118409 |
|  | 12 | RCT | 42369 |
|  | 13 | #1 OR #2 OR #3 OR #4 | 2184 |
|  | 14 | #5 OR #7 OR #8 OR #9 OR #10 OR #11 | 43209 |

|  | 15 | #13 OR #14 | 1123719 |
| --- | --- | --- | --- |
|  | 16 | #5 AND #12 AND #15 | 633 |
| ClinicalTrial s.gov websites | 1 | ticagrelor | 377 |
