# Supplementary material for: The effect of ticagrelor on coronary microvascular function after PCI in patients with ACS compared to clopidogrel: A systematic review and meta-analysis
Source: PLoS One. 2023 Aug 29;18(8):e0289243. doi: 10.1371/journal.pone.0289243 (PMC10464986; doi:10.1371/journal.pone.0289243)
Supplement: S2 Table — (DOCX) [file pone.0289243.s012.docx]

| outcomes | Summary of findings | | | Quality assessment | |  |  |  |  | Certainty of evidence |
| --- | --- | --- | --- | --- | --- | --- | --- | --- | --- | --- |
|  | No.stud | RR(95%CI) | MD(95%CI) | Study design | Risk of bias | Inconsisten | Indirectne | Imprecision | Other considerat |  |
| IMR | 4 | - | -6.23(-8.41,-4.04) | randomised  trials | serious | not serious | not seriou | not serious | none | ⨁⨁⨁⭕ Moderate |
| CFR | 4 | - | 0.38(0.18,0.57) | randomised  trials | serious | not serious | not seriou | serious | none | ⨁⨁⭕ ⭕ Low |
| MBG | 4 | 1.29(1.12,1.48) | - | randomised  trials | serious | serious | not seriou | not serious | none | ⨁⨁⭕ ⭕ Low |
| TIMI | 12 | 1.03(1.00,1.06) | - | randomised  trials | serious | not serious | not seriou | serious | none | ⨁⨁⭕ ⭕ Low |
| cTFC | 5 | - | -2.09(-2.93,-1.24) | randomised  trials | serious | serious | not seriou | serious | none | ⨁⨁⭕ ⭕ Low |
