# Supplementary material for: The effect of ticagrelor on coronary microvascular function after PCI in patients with ACS compared to clopidogrel: A systematic review and meta-analysis
Source: PLoS One. 2023 Aug 29;18(8):e0289243. doi: 10.1371/journal.pone.0289243 (PMC10464986; doi:10.1371/journal.pone.0289243)
Supplement: S1 File — (DOCX) [file pone.0289243.s013.docx]

Included study

1. Xu J, Lo S, Mussap CJ, French JK, Rajaratnam R, Kadappu K, Premawardhana U, Nguyen P, Juergens CP, Leung DY. Impact of Ticagrelor Versus Clopidogrel on Coronary Microvascular Function After Non-ST-Segment-Elevation Acute Coronary Syndrome. Circ Cardiovasc Interv. 2022 Apr;15(4):e011419. doi: 10.1161/CIRCINTERVENTIONS.121.011419.
2. Park K, Cho YR, Park JS, Park TH, Kim MH, Kim YD. Comparison of the Effects of Ticagrelor and Clopidogrel on Microvascular Dysfunction in Patients with Acute Coronary Syndrome Using Invasive Physiologic Indices. Circ Cardiovasc Interv.2019Oct;12(10): e008105.doi:10.1 161/ CIRCINTERVENTIONS.119.008105.
3. Park SD, Lee MJ, Baek YS, Kwon SW, Shin SH, Woo SI, Kim DH, Kwan J, Park KS. Randomised trial to compare a protective effect of Clopidogrel Versus TIcagrelor on coronary Microvascular injury in ST-segment Elevation myocardial infarction (CV-TIME trial). EuroIntervention. 2016 Oct 10;12(8):e964-e971. doi: 10.4244/EIJV12I8A159.
4. Woong Gil Choi.Abstract 12019: Differential Effect of Ticagrelor versus Clopidogrel on Coronary Microvascular Function in Patients With Coronary Aertery Disease.Circulation. 2014;130: A12019
5. Winter JL, Lindefjeld DS, Veas N, Guarda E, Valdebenito M, Méndez M, Pérez O, Zuanic K, Mestas M, Martínez A. Angiographic and electrocardiographic parameters of myocardial reperfusion in angioplasty of patients with ST elevation acute myocardial infarction loaded with ticagrelor or clopidogrel (MICAMI-TICLO trial). Cardiovasc Revasc Med. 2014 Jul-Aug;15(5):284-8. doi: 10.1016/j.carrev.2014.07.001. Epub 2014 Jul 12.
6. Mont'Alverne-Filho JR, Rodrigues-Sobrinho CR, Medeiros F, Falcão FC, Falcão JL, Silva RC, Croce KJ, Nicolau JC, Valgimigli M, Serruys PW, Lemos PA. Upstream clopidogrel, prasugrel, or ticagrelor for patients treated with primary angioplasty: Results of an angiographic randomized pilot study. Catheter Cardiovasc Interv. 2016 Jun;87(7):1187-93. doi: 10.1002/ccd.26334. Epub 2015 Nov 28.
7. Di Vito L, Versaci F, Limbruno U, Pawlowski T, Gatto L, Romagnoli E, Cattabiani MA, Micari A, Trivisonno A, Marco V, Prati F. Impact of oral P2Y12 inhibitors on residual thrombus burden and reperfusion indexes in patients with ST-segment elevation myocardial infarction. J Cardiovasc Med (Hagerstown). 2016 Sep;17(9):701-6. doi: 10.2459/JCM.0000000000000392.
8. Wang X, Li X, Wu H, Li R, Liu H, Wang L, Bai S, Zhang L, Chen T, Liu J, Li Q, Du R. Beneficial effect of ticagrelor on microvascular perfusion in patients with ST-segment elevation myocardial infarction undergoing a primary percutaneous coronary intervention. Coron Artery Dis. 2019 Aug;30(5):317-322. doi: 10.1097/MCA.0000000000000707.
9. Cao B, Qu F, Liu X, Gao C, Fu Q, Jiang C, Wei P, Ma Q. Short-term efficacy of ticagrelor in acute ST-segment elevation myocardial infarction patients undergoing an emergency percutaneous coronary intervention. Aging (Albany NY). 2019 Oct 30;11(20):8925-8936. doi: 10.18632/aging.102353. Epub 2019 Oct 30.
10. Kim EK, Park TK, Yang JH, Song YB, Choi JH, Choi SH, Chun WJ, Choe YH, Gwon HC, Hahn JY. Ticagrelor Versus Clopidogrel on Myocardial Infarct Size in Patients Undergoing Primary Percutaneous Coronary Intervention. J Am Coll Cardiol. 2017 Apr 25;69(16):2098-2099. doi: 10.1016/j.jacc.2017.02.034.
11. Zhu K, Fu Q, Zhang N, Huang YJ, Zhang Q. Pre-PCI medication using clopidogrel and ticagrelor in the treatment of patients with acute myocardial infarction. Eur Rev Med Pharmacol Sci. 2015 Dec;19(23):4636-41.
12. Petousis S, Hamilos M, Pagonidis K, Vardas P, Lazopoulos G, Anastasiou I, Zacharis E, Kochiadakis G, Skalidis E. Assessment of myocardial salvage in patients with STEMI undergoing thrombolysis: ticagrelor versus clopidogrel. BMC Cardiovasc Disord. 2022 Jul 2;22(1):301. doi: 10.1186/s12872-022-02735-1.
13. Kunadian V, James SK, Wojdyla DM, Zorkun C, Wu J, Storey RF, Steg PG, Katus H, Emanuelsson H, Horrow J, Maya J, Wallentin L, Harrington RA, Gibson CM. Angiographic outcomes in the PLATO Trial (Platelet Inhibition and Patient Outcomes). JACC Cardiovasc Interv. 2013 Jul;6(7):671-83. doi: 10.1016/j.jcin.2013.03.014.
14. Liu Y, Ding LY, Li XZ. Therapy with ticagrelor for ST-elevated acute coronary syndrome accompanied by diabetes mellitus. Eur Rev Med Pharmacol Sci. 2019 Aug;23(3 Suppl):312-318. doi: 10.26355/eurrev_201908_18662.
15. Liu Y, Liu H, Hao Y, Hao Z, Geng G, Han W, Chen Q, Wang D, Liu L, Jia K, Zhou Y. Short-term efficacy and safety of three different antiplatelet regimens in diabetic patients treated with primary percutaneous coronary intervention: a randomised study. Kardiol Pol. 2017;75(9):850-858. doi: 10.5603/KP.a2017.0116. Epub 2017 Jun 14.
16. Hamilos M, Kanakakis J, Anastasiou I, Karvounis C, Vasilikos V, Goudevenos J, Michalis L, Koutouzis M, Tsiafoutis I, Raisakis K, Stakos D, Hahalis G, Vardas P; Collaborators. Ticagrelor versus clopidogrel in patients with STEMI treated with thrombolysis: the MIRTOS trial. EuroIntervention. 2021 Feb 19;16(14):1163-1169. doi: 10.4244/EIJ-D-20-00268.
